# Differentiated Thyroid Carcinoma Long-Term Prognostic Factors

**DOI:** 10.1155/2024/1067447

**Published:** 2024-09-10

**Authors:** Pacheco-Ojeda Luis, Martínez-Jaramillo Ana Lucía, Romo-Castillo Hugo, Recalde-Maldonado Ramiro, Cañizares-Quisiguiña Stalin

**Affiliations:** ^1^ Surgery Service Metropolitano Hospital, Quito, Ecuador; ^2^ Endocrinology Service Specialities Hospital Carlos Andrade Marín, Quito, Ecuador; ^3^ Faculty of Medical Sciences Central University, Quito, Ecuador; ^4^ San Francisco de Quito University, Quito, Ecuador

## Abstract

**Introduction:**

Thyroid cancer is the most common cancer in women in Ecuador.

**Objective:**

The aim of this study was to determine the demographics and clinical and treatment variables of patients with papillary or follicular thyroid cancer, referred to as differentiated thyroid cancer (DTC), treated at a third-level hospital in Quito, Ecuador.

**Methods:**

We reviewed retrospectively the medical records of patients with DTC, who underwent surgical treatment, from 1990 to 2019. Data included demographics, pathological information, clinical stage, type of surgery, and radioactive iodine (RAI) adjuvant therapy. Patients were monitored for up to 29 years (median follow-up time 6.9 years).

**Results:**

The corrected overall 5-, 10-, 20-, and 30-year survival rates (Kaplan–Meier) were 93%, 85%, 70%, and 63%, respectively. On univariate analysis, age, histological type, tumor grade, histological variants, capsular invasion, vascular invasion, tumor size, clinical stage, distant metastases at diagnosis, surgical margins, extrathyroidal invasion, radioactive iodine adjuvant treatment, and locoregional recurrence were found to be significant prognostic factors. In a multivariate analysis, the following independent variables: age over 55 years, extrathyroidal spread, metastasis at diagnosis, and stage II to IV raised the risk of death (hazard risk) (HR).

**Conclusions:**

Age over 55 years, extrathyroidal spread, metastasis at diagnosis, and advanced clinical stage were found to have a harmful prognosis and an increased risk of death in a series of Ecuadorian patients surgically treated for a DTC.

## 1. Introduction

Thyroid cancer is the commonest endocrine malignancy. A steadily increasing incidence has been observed in developed as well as developing countries [1]. In the United States, in 2018, the incidence in men and women was 3.4 and 10.1 per 100.000 inhabitants, respectively. In Ecuador, the current incidence is 8.2 and 40.9 for both sexes [2]. This incidence in Ecuadorian women is the fifth highest in the world. On the other hand, mortality in men and women has remained low: 0.34 and 0.48 per 100.000 inhabitants in the United States [1] and 1.1 and 2.7, respectively, in Ecuador [2].

Prognostic factors have been intensively studied since the 1980s. To predict outcomes and select individualized treatment, numerous staging classification systems were proposed such as AMES, GAMES, and MACIS. The American Thyroid Association (ATA) published in 2016 an initial risk stratification system for thyroid carcinoma (DTC) utilizing prognostic factors such as histologic type, pathology characteristics, and mutational status to assist the decision-making for radioactive I^131^ adjuvant treatment [3].

The aim of this study was to determine the predictive of demographics and clinical and treatment variables of patients with papillary or follicular thyroid cancer, referred to as differentiated thyroid cancer (DTC), treated at a third-level hospital in Quito, Ecuador, South America.

## 2. Materials and Methods

We reviewed retrospectively the medical records of patients with DTC, who underwent surgical treatment, from 1990 to 2019 at a tertiary public hospital in Quito, Ecuador. All patients signed an informed consent before surgery. The hospital's ethics committee permission was obtained. Data included demographics, pathological information, clinical stage, type of surgical procedure, and radioactive iodine (RAI) adjuvant therapy. Patients were followed up to 29 years with a median time of 6.9 years. Pathological diagnoses and clinical staging were based on the Protocol for the Examination of Specimens from Patients with Carcinomas of the Thyroid Gland of the American College of Pathologists and the AJCC [4, 5].

### 2.1. Statistical Analysis

Continuous variables are summarized as mean ± SD and categorical variables as percentages. Age was divided into two groups, age ≤55 years and age >55 years. Patients were stratified by using the TNM staging criteria.

To assess survival rates, we employed the univariate analysis with Kaplan–Meier and the log-rank test to compare two or more survival curves of unadjusted overall survival between categorical variables and death. For multivariate analysis, we used the semiparametric Cox proportional hazards model to identify risk factors for mortality. *P* value with a significance level set at *P* < 0.05, adjusted hazard ratio (HR), and 95% confidence interval (CI) are also reported.

The best-fitting Cox proportional hazards model was selected using the Akaike information criteria (AIC). The final model had only four covariates that satisfied the proportionality hazard assumption, namely, age over 55 years, carcinoma stage, compromised margins, and extrathyroidal extension. The model was assessed with Schoenfeld's global test to assess the proportional hazards assumption in the Cox model.

Adjusted HR and 95% CI are reported. Data analyses were performed using RStudio, version 1.4.1106 [6].

## 3. Results

### 3.1. Patient Demographics and Clinical Characteristics

From 1990 to 2019, a total of 875 patients with either follicular or papillary thyroid cancer were identified. Of those, there were 839 (96%) patients with papillary cancer and 36 (4%) patients with follicular thyroid cancer. A total of 734 (84%) patients were women and 606 (69%) were older than 55 years (Table 1). In the univariate analysis, all covariates were statistically significant in age, sex, type of tumor, and type of surgery between groups. Thyroid surgery was divided between total/subtotal thyroidectomy (*n* = 828 patients (95%)) and partial (lobectomy) thyroidectomy (*n* = 47 patients (5%)). Of most patients, 765 (87%) had a stage I tumor, 58 (7%) had a stage II tumor, 38 (4%) had a stage III tumor, and 14 (2%) had a stage IV tumor (Table 1).

### 3.2. Survival and Prognostic Factors' Analysis

The corrected overall 5-, 10-, 20-, and 30-year survival rates (Kaplan–Meier) were 93%, 85%, 70%, and 63%, respectively.

Seventeen features considered for univariate analysis of survival are shown in Table 2. Sclerosing, tall cells, columnar cells, and insular carcinomas were considered aggressive histological variants.

On univariate analysis, age, histological type, tumor grade, histological variants, capsular invasion, vascular invasion, tumor size, clinical stage, distant metastases at diagnosis, surgical margins, extrathyroidal invasion, radioactive iodine adjuvant treatment, and locoregional recurrence were found to be significant prognostic factors.

### 3.3. Cox Proportional Hazards Model

**Figure 1:**
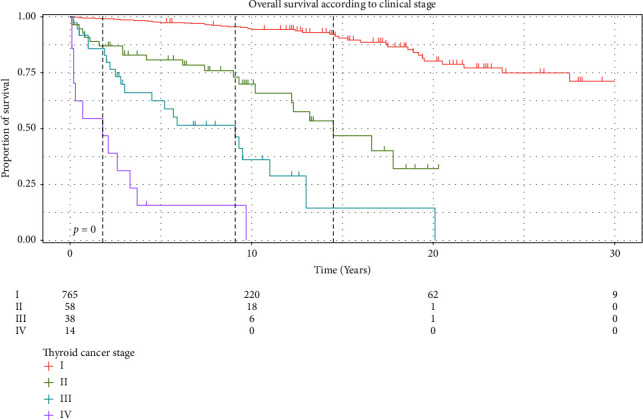
Overall survival according to clinical stage.

In the multivariate analysis, Cox PH model, beta regression coefficients from all covariates included in the model reached a high statistical significance. Though, having death as the dependent variable, the independent variables such as age over 55 years, extrathyroidal spread, the presence of metastasis at presentation and stage II to IV tumors of the thyroid cancer raised the hazard risk of death (HR) (Figure 1). The exponentiated coefficients or hazard ratios of those covariates gave us the effect size. On the other hand, the global significance of the model tested by asymptotically equivalent tests such as the Schoenfeld global test, likelihood ratio, Wald test, and score log-rank statistics confirmed model significance.

Since the requirement of the Cox PH model is the inclusion of covariates in the dataset that satisfy the proportional hazard (PH) assumption, we used the Schoenfeld residual test in RStudio to make sure that regression parameters were constant over time. Covariates that did not satisfy the PH assumption were excluded from the final Cox PH model. Results from this analysis are presented in Table 3.

According to the hazard ratio obtained, patients with thyroid carcinoma older than 55 years old, at a given instant in time are 3.32 times as likely to die as those who are younger than 55 years old, while keeping constant other explicative variables (*p* < 0.0001). Using the Akaike information criteria (AIC), the best-fitting Cox PH model had the four covariates that fit the proportional hazard Cox model assumptions. The histological type of thyroid cancer, compromised margins, lymph node involvement, tumor size, and type of surgery neither reached significance nor improved the model prediction, therefore, those variables were excluded. Meanwhile, age, thyroid cancer stage, and metastasis plus extrathyroidal extension did fit the model and they were selected by stepwise approach. The thyroid carcinoma stage IV patients had a HR = 6.19, 95% CI (1.09–35.04), and *p*=0.05 of worse survival compared to patients in stage I adjusted to other covariates.

By contrast, the follicular type of cancer that in the univariate analysis had a worse prognosis than papillary carcinoma, once included in the model and holding the other covariates constant, their *p* value was not significant. Having fit a Cox model to the data, we visualized the predicted survival proportion at any given point in time for a particular risk group and estimated the survival proportion, at the mean values of covariates. The following plot shows a fairly good predicted survival probability for our series of thyroid cancer patients surgically treated and followed up longer than 40 years (Table 4).

## 4. Discussion

DTC has a good prognosis in most cases. However, some patients may have some characteristics that can determine an adverse outcome.

Even if age, histological type, tumor grade, histological variants, capsular invasion, vascular invasion, tumor size, clinical stage, distant metastases at diagnosis, surgical margins, extrathyroidal invasion, radioactive iodine adjuvant treatment, and locoregional invasion were found to be significantly prognostic by univariate analysis, only age >55 years, extrathyroidal spread, the presence of metastasis at diagnosis, and tumors stratified into stages II to IV of the AJCC Staging Classification System remained as independent significant prognostic factors by multivariate analysis in the present study.

In a recent series of 422 thyroid cancer cases, age, initial lymph node involvement, number of radioiodine therapies, and histopathology of the tumor were selected as independent significant predictors for mortality [7]. The most unfavorable factors of the prognosis for patients with DTC in a series of 5526 patients reported by Guda [8] were stage IV A and age older than 60. Other prognostic factors (multifocal tumor growth, lymph node involvement, male sex, and recurrence) were also predictive factors, but with a somewhat less significance.

In another series of 6015 papillary thyroid carcinoma reported by Ito [9], the important prognostic factors were age 55 years or older, distant metastasis at surgery, clinical lymph node metastasis measuring 3 cm or larger, extranodal tumor extension, and significant extrathyroidal extension. Tumors larger than 4 cm, clinical node metastasis smaller than 3 cm with no extranodal tumor extension, and male gender were moderate prognostic factors. In this large series report, on multivariate study, age at 55 years or older was the most significant prognostic factor for cause-specific survival (CSS), except for distant metastasis at surgery [9]. Age was a significant independent prognostic factor, by multivariate analysis, in the present series.

Regarding tumor histology, the 10-year survival for papillary thyroid cancer (PTC) is around 95% and for follicular thyroid cancer (FTC), 70 to 95%. This slightly worse survival for FTC has been described to be possibly due to later presentation and the presence of distant metastases at diagnosis [10]. Even if PTC had a significantly better prognosis in our patients, by univariate analysis, it was not confirmed by multivariate analysis in the present series.

In a systematic review with meta-analysis, Kim [11] found that multifocality was significantly associated with an increased risk of recurrence, while cancer-specific survival showed no difference. In subgroup analyses, the hazard ratios of multifocality for recurrence were associated with primary tumor size (1.81 and 1.90 for 1 cm versus >1 cm, respectively), number of tumor foci (1.45 and 1.95 for 2 foci versus 3 foci, respectively), and patient age (HRs for pediatric and adult patients were 3.19 and 1.89 for pediatric versus adult patients, respectively). Multifocality was not a significant prognostic factor in our study.

The presence of tumor capsular invasion has appeared not to have significance for the long-term prognosis of patients with PTC or FTC since early studies [12]. Encapsulated tumors with microscopic capsular invasion are currently considered as minimally invasive [3]. On the other hand, a recent meta-analysis demonstrated a significant impact of vascular invasion on tumor recurrence and patient survival in DTC patients [13], so the authors recommended considering the presence and extent of vascular invasion as an adverse prognostic factor in DTC. In a large study with patients registered in the National Cancer Database of the United States of America [14], it was demonstrated that the presence of lymphovascular invasion among patients with PTC was independently associated with compromised overall survival. It was concluded that these patients should be considered at higher risk, and adjuvant RAI should be more strongly considered. In our study, on univariate analysis, vascular invasion appeared as a very significant prognostic factor, capsular invasion had only mild significance, and lymphatic invasion was not significant.

The combined aggressive pattern carcinomas, when compared with the other variants, classical and follicular, had a higher risk of invasion of the thyroid capsule invasion, lymphovascular invasion, extrathyroid invasion, and lymph node metastasis. These aggressive variants are also associated with higher rates of recurrence and metastasis and may have lower survival rates [15]. In a large multi-institutional study including 91,145 patients from the National Cancer Database, Khokar [16] reported that tall cell variants had worse overall survival than classical and diffuse sclerosing variants which had both similar survivals. In another recent large study, Xu [17] reported a significantly highest 5 year overall and disease-specific survival for the follicular variant, followed by the conventional variant and by tall cell variant.

Staging is important not only to predict outcomes but also to facilitate treatment decision-making [18]. In the present study, we found, by multivariate analysis, that stages II to IV significantly raised the risk of death. Stage I (microcarcinoma, <1 cm in size) patients had an excellent 95% 10-year survival.

Distant metastasis at diagnosis is one of the most important prognostic factors for cause-specific survival (CSS) of patients. They are more likely found in patients showing aggressive behavior and directly linked to other clinicopathological features such as gender, tumor size, and extrathyroidal extension [9]. In our study, distant metastases at the time of diagnosis appeared to be an independent prognostic factor, by multivariate analysis.

Recent data have demonstrated that in properly selected patients, clinical outcomes are very similar following unilateral or bilateral thyroid surgery [3, 10]. Our finding that the partial and total thyroidectomy do not differ significantly in survival would indicate that a more adapted surgical procedure based on tumor size end extent and lymph node involvement is required.

A meta-analysis based on six studies with 7696 patients did not find a statistically significant association between microscopically positive surgical margins and local recurrence. So, a finding of microscopically positive surgical margin in the absence of other adverse factors would not be an indication for adjuvant treatment [19]. However, there is controversy regarding the prognostic value of microscopic extrathyroidal tumor extension (MEE) [20]. Positive surgical margins were not a prognostic factor in our study.

Macroscopic extrathyroidal extension on intraoperative evaluation is an important factor in predicting a worse prognosis for patients with PTC. However, three categories of this extension, each with a different prognosis, were defined in the 8^th^ edition of the AJCC staging system: T3b tumors, invading only the strap muscles; T4a tumors invading subcutaneous soft tissues, larynx, trachea, esophagus, or recurrent laryngeal nerve; and T4 tumors invading the prevertebral fascia or encasing the carotid artery or mediastinal vessels [5]. In our study, extrathyroidal extension was an important prognostic factor by multivariate analysis.

Multiple studies have reported no association between regional lymph node metastases and overall survival but a consistent correlation with local recurrence has been described [7, 18, 21–23]. We did not find a statistical influence of pathologically positive lymph nodes on survival.

The use of I131 treatment after thyroidectomy improves clinical outcomes in terms of recurrence and survival in selected patients with DTC. This benefit has been observed in advanced disease stages but not in small primary tumors [10]. According to the ATA guidelines, RAI remnant ablation after thyroidectomy is not recommended for low-risk DTC patients, and it should be considered for intermediate-risk patients and recommended to high/risk patients. Even if RAI adjuvant treatment improved survival in the whole cohort of patients in our series, by univariate analysis, it was not found effective by multivariate analysis.

The impact of locoregional recurrences on the long-term survival of patients with PTC has been rarely discussed in the literature. In the present series, patients with locoregional recurrences had a significantly compromised overall survival. In a study with data collected from 1636 subjects with PTC at National Taiwan University Hospital [24], the locoregional recurrences had a moderately harmful impact on overall and disease-specific survival.

An analysis of the prognostic impact of other demographics [25], pathological, clinical, and therapeutic variables, as well as the study of the influence of additional factors within each variable, analyzed in the present cohort of patients, is warranted in the future.

One strength in this series is that follow-up was performed in all our patients, even if it was not long enough in a group of them, a fact that is difficult to obtain in Latin America. Only a few studies in this region had included follow-up results [26, 27]. However, limitations include the retrospective nature of the study and the lack of digital data in the patients treated in the first years of this study.

## 5. Conclusions

Overall long-term survival of a series of Ecuadorian patients with DTC and surgically treated has been as good as in all reported data. However, some factors such as an age >55 years old, extrathyroidal spread, the presence of metastasis at diagnosis, and advanced clinical stage were found to have a less good prognosis and an increased risk of death, by multivariate analysis.

## Figures and Tables

**Table 1:** Demographic and clinical characteristics of the population.

Features	*n* (%)
	875

*Age (y), mean* *±* *SD*	47.82 ± 14.28
≤55 y	606 (69.3%)
>55 y	269 (30.7%)

*Histology*	
Papillary	839 (95.9%)
Follicular	36 (4.1%)

*Multifocality*	
Yes	301 (34.4%)
No	574 (65.6%)

*Tumor grade*	
Well-differentiated	308 (55.6%)
Moderately-differentiated	230 (41.52%)
Poorly-differentiated	16 (2.89%)

*Capsular invasion*	
Yes	495 (63.38%)
No	286 (36.62%)

*Vascular invasion*	
Yes	184 (36.29%)
No	323 (63.71%)

*Lymphatic invasion*	
Yes	131 (34.29%)
No	251 (65.71%)

*Aggressive variants*	
Yes	94 (19.46%)
No	389 (80.54%)

*Tumor size*	
≥1 cm (pT1a)	211 (24.1%)
1–4 cm (p1Tb–T3b)	573 (65.5%)
>4 cm (T4a–T4b)	91 (10.4%)

*Distant metastasis at presentation*	
Yes	18 (2.1%)
No	857 (97.9%)

*Stage*	
I	765 (87.4%)
II	58 (6.6%)
III	38 (4.3%)
IV	14 (1.6%)

*Type of thyroidectomy*	
Total/near total	828 (94.6%)
Partial	47 (5.4%)

*Surgical margins*	
Clear	497 (56.8%)
Close or positive	329 (37.6%)
No reported	49 (5.6%)

*Extrathyroidal invasion*	
Yes	173 (19.8%)
No	702 (80.2%)

*Lymph node involvement*	
Yes	368 (42.1%)
No	507 (57.9%)

*Adjuvant iodine treatment*	
Yes	621 (71.0%)
No	254 (29.0%)

*Locoregional recurrence*	
Yes	137 (15.66%)
No	738 (84.34%)

**Table 2:** Overall survival by univariate analysis.

Features	5-year Survival p	10-year Survival p	20-year Survival p	30-year Survival p	*P* value
*Age (y), mean* *±* *SD*					
≤55 y	96.9	96.2	83.3	—	<0.0001
>55 y	84.5	68.9	34.5	—	

*Histology*					
Papillary	93.9	89.7	73.6	—	<0.0001
Follicular	76.4	52.6	23.0	—	

*Multifocality*					
Yes	92.57	88.12	68.34	—	0.87
No	92.52	86.59	71.8	—	

*Tumor grade*					
Well-differentiated	94.43	92.43	83.83	—	
Moderately-differentiated	94.6	86.18	62.78	—	<0.0001
Poorly-differentiated	43.8	43.8	43.8	—	

*Capsular invasion*					
Yes	90.86	85.73	71.97	61.64	0.026
No	96.39	92.04	65.49	65.49	

*Vascular invasion*					
Yes	85.9	77.1	53.2	—	0.00011
No	95.22	89.6	78.77	—	

*Lymphatic invasion*					
Yes	91.8	86.19	79.01	—	0.12
No	95.57	90.12	75.1	—	

*Aggressive variants*					
Yes	87.05	81.52	—	—	0.0061
No	94.33	90.2	—	—	

*Tumor size*					
≥1 cm (pT1a)	96.6	96.5	71.6	—	
1–4 cm (p1Tb-T3b)	93.9	91.7	74.7	—	<0.0001
>4 cm (T4a-T4b)	76.4	69.2	56.0	—	

*Distant metastasis at presentation*					
Yes	24.1	12.1		—	<0.0001
No	94.8	89.8	73.1	—	

*Stage*					
I	97.4	94.6	—		
II	81.0	67.3	—		<0.0001
III	59.9	36.7	—		
IV	16.1	—	—		

*Type of thyroidectomy*					
Total/near total	93.2	88.2	75.7	67.0	0.8110
Partial	91.29	84.1	68.9	56.9	

*Surgical margins*					
Clear	94.9	90.7	73.9	66.0	
Close or positive	93.0	82.7	65.1	—	0.0037
No reported					

*Extrathyroidal invasion*					
Yes	81.5	75.3	7.3	—	<0.0001
No	96.2	91.0	75.4	—	

*Lymph node involvement*					
Yes	90.2	74.2	70.0	—	0.0618
No	82.2	64.8	62.8	—	

*Adjuvant iodine treatment*					
Yes	95.9	90.7	72.3	—	<0.0001
No	84.6	78.1	61.5	—	

*Locoregional recurrence*					
Yes	86.7	75.03	48.65	35.7	<0.0001
No	93.91	90.23	79.35	73.68	

**Table 3:** Risk factors for mortality in thyroid cancer.

Covariate/level	Univariate Cox regression			Multivariate Cox regression		
	Hazard ratio	CI 95%	*P* value	Hazard ratio	CI 95%	*P* value
Age >55 years	7.28	4.65–11.40	<0.0001	3.17	1.62–6.21	<0.0007
Gender (males)	0.64	0.39–1.03	0.07	0.87	0.53–1.46	0.6117
Histology (papillary)	0.20	0.12–0.36	<0.0001	0.81	0.41–1.64	0.5694
T-stage						
I	Reference					
II	7.25	4.25–12.37	<0.0001	2.24	0.99–5.06	0.0528
III	17.59	10.25–30.20	<0.0001	6.52	2.69–15.77	0.0003
IV	67.69	33.98–134.83	<0.0001	3.30	0.48–22.49	0.2226
Tumor size						
<1 cm	Reference					
1–4 cm	1.44	0.71–2.90	0.30	1.02	0.48–2.13	0.9651
>4 cm	4.96	2.50–9.82	<0.0001	1.24	0.55–2.82	0.6044
Surgery						
Lobectomy	Reference					
Total/subtotal ref	0.92	0.48–1.77	0.8	1.13	0.55–2.29	0.7429
Extrathyroidal invasion (yes)	3.39	2.27–5.06	<0.0001	1.55	0.86–2.79	0.1417
Lymph node invasion (yes)	0.67	0.44–1.02	0.06	0.93	0.58–1.49	0.7632
Margin invasion (yes)	1.79	120−2.68	0.0004	1.24	0.78–1.98	0.3695
Metastasis (yes)	19.71	10.91–35.6	<0.0001	7.23	1.60–32.57	0.0099
RAI (yes)	0.41	0.27–0.61	<0.0001	0.37	0.23–0.61	<0.0001

**Table 4:** Cox proportional hazards model selected.

Covariates	Hazard ratio^1^	95% CI^1^	*p* value
Age >55 y.o.	3.32	1.78, 6.22	<0.0002
Stage			
I	Reference		
II	2.77	1.40, 5.47	0.0032
III	5.62	2.62, 12.05	<0.0001
IV	6.19	1.09, 35.04	0.0567
Extrathyroidal invasion (yes)	1.75	1.05, 2.90	0.0308
Metastasis (yes)	4.84	1.13, 20.82	0.0342

^1^HR, hazard ratio. CI, confidence interval concordance = 0.84 (SE = 0.026); LR test = 174.7; Wald test = 196.4; score (log-rank) test = 473.2, 6 *dfp* < 0.0001.

## Data Availability

The data to support the findings of this study is available on request from the Carlos Andrade Marín Hospital IMB (AS400) software system.
